# Multimodality Image Fusion with PSMA PET/CT and High-Intensity Focused Ultrasound Focal Therapy for Primary Diagnosis and Management of Prostate Cancer: A Planned Research Initiative

**DOI:** 10.5041/RMMJ.10312

**Published:** 2017-10-16

**Authors:** Marcia C. Javitt, Alexander Kravtsov, Zohar Keidar, Sobhi Abadi, Gilad E. Amiel

**Affiliations:** 1 Department of Medical Imaging, Rambam Health Care Campus, Haifa, Israel; 2 Department of Urology, Rambam Health Care Campus, Haifa, Israel; 3 Department of Nuclear Medicine, Rambam Health Care Campus, Haifa, Israel

**Keywords:** Fusion, high-intensity focused ultrasound focal therapy, MRI, PSMA PET/CT, prostate cancer, TRUS

## Abstract

Recent developments in diagnostic imaging herald a new approach to diagnosis and management of prostate cancer. Multimodality fusion that combines anatomic with functional imaging data has surpassed either of the two alone. This opens up the possibility to “find and fix” malignancy with greater accuracy than ever before. This is particularly important for prostate cancer because it is the most common male cancer in most developed countries. This article describes technical advances under investigation at our institution and others using multimodality image fusion of magnetic resonance imaging (MRI), transrectal ultrasound (TRUS), and PSMA PET/CT (defined as the combination of prostate-specific membrane antigen [PSMA], positron emission tomography [PET], and computed tomography [CT]) for personalized medicine in the diagnosis and focal therapy of prostate cancer with high-intensity focused ultrasound (HiFUS).

## INTRODUCTION

In the United States, prostate cancer is the third most common cause of cancer death, with 161,360 new cases and 26,730 deaths expected in 2017.^1^ Worldwide 1,111,700 cases and 307,500 deaths are attributed to prostate cancer.^2^ While the incidence of prostate cancer spiked from about 1980 to 2000 due to prostate-specific antigen (PSA) blood test screening, recently the rates have declined dramatically because screening with PSA is no longer recommended for men of average risk.^3^ This is largely due to concerns about overdiagnosis and significant morbidity associated with overtreatment of prostate cancer. Overdiagnosis and overtreatment are estimated to occur in up to 42% of cases.^3^,^4^ There are also concerns about underdiagnosis of clinically significant high-risk cancers.^3^,^4^ In other words, currently there are no biomarkers that enable cost-effective screening for prostate cancer in average-risk patients.

At present, transrectal ultrasound (TRUS) with saturation biopsies is the standard of care for detection of prostate cancer, but it is limited due to sampling errors, overdetection of clinically insignificant cancers, and underdetection of some aggressive lesions. Multiparametric magnetic resonance imaging (mpMRI) combines anatomic and functional data, is routinely used for lesion detection and characterization, and enables risk stratification. However, MRI interpretation is labor-intensive, requiring significant expertise. Because positron emission tomography (PET) takes advantage of cell metabolism, cell division, and receptor binding to depict functional processes with well-known high sensitivity, new receptor-targeted agents for imaging prostate cancer (such as prostate-specific membrane antigen, PSMA) can add high specificity.

How can we take advantage of the complementary information provided by these different imaging modalities? Combining them for a single unified interpretation is now feasible using image fusion and hybrid techniques. This report will focus on the fusion of ultrasound (US), MRI, and PSMA PET/CT to detect clinically significant organ-confined prostate cancers, biopsy these target lesions, and institute focal therapy. The situation is analogous to the adoption of lumpectomy and radiation as breast-conserving surgery for breast cancer that occurred in the late 1980s in place of radical mastectomy in women.^5^ By sparing normal tissue, this “male lumpectomy” (i.e. focal therapy) reduces the risk of complications and accelerates recovery. One such focal therapy is high-intensity focused ultrasound (HiFUS).

This article describes the mandate for our institution’s interdepartmental research plan. We plan to employ multimodality image fusion for precise diagnosis, local staging, and focal therapy of organ-confined prostate cancer using HiFUS. Looking further forward, future multi-center randomized controlled trials comparing focal therapy to standard treatment with radical prostatectomy are needed to document equivalence or superiority of focal therapy before its widespread adoption can be recommended.

## IMAGING METHODS

### Ultrasound

**Figure 1:**
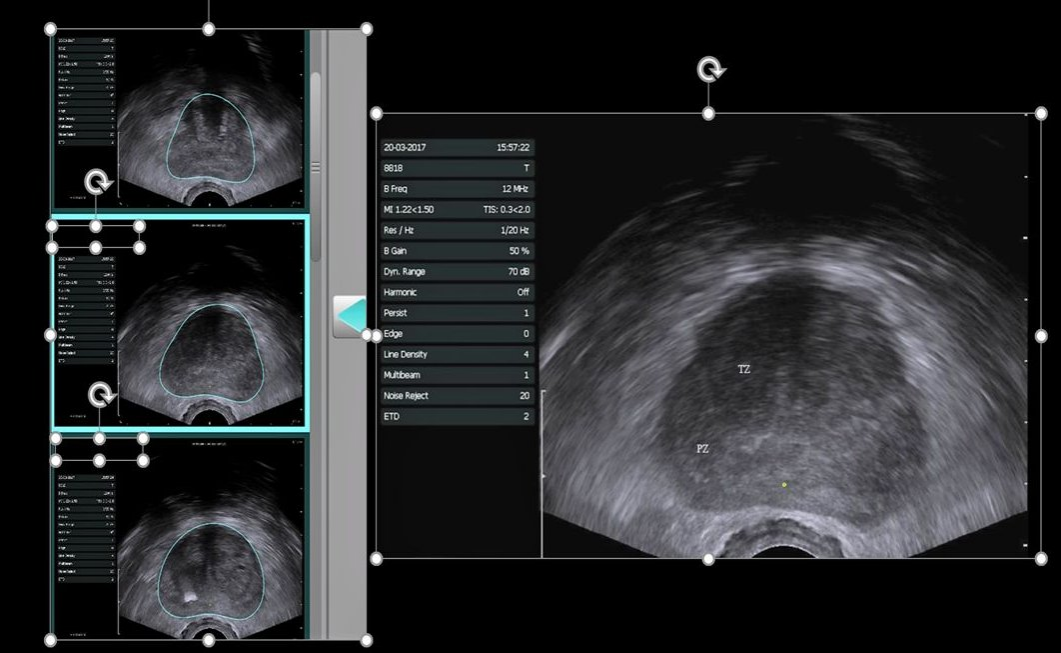
Transrectal Ultrasound Normal TRUS in axial plane shows transitional zone (TZ) and peripheral zone (PZ) without focal hypoechoic nodules. Scanning is volumetric and can be depicted in all three planes: axial, sagittal, and coronal.

**Figure 2:**
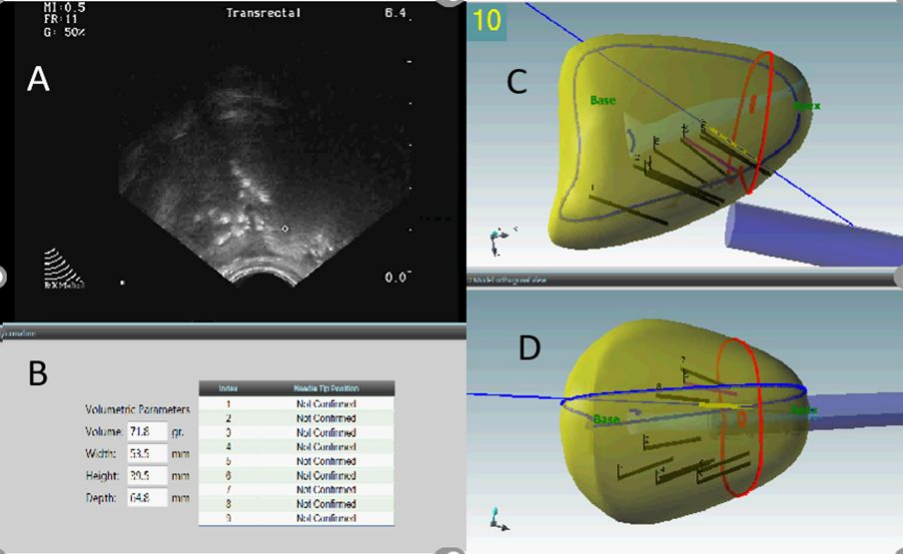
3D TRUS-Guided Prostate Biopsy in a 47-Year-Old Male Uses 3D Prostate Model Transrectal US of the prostate (A) is used to identify suspicious areas and to perform systematic saturation biopsies. Note the cursor in (A) overlying a focal round nodule that was biopsied. The volume of the gland is automatically calculated using measurements of width, height, and depth applied to the formula of a prolate ellipse volume (height × length × width × π/6). Biopsies are performed using the 3D volumetric rendering of the gland (C and D) with mapped record of all passes into the prostate.

Transrectal US (Figure 1) with systematic biopsies combined with targeting of any focal hypoechoic foci is the standard of care for diagnosis of prostate cancer. Transrectal US is performed in real time for guidance of needle position and throw using needle tracking (Figure 2). Transrectal US has no ionizing radiation, wide availability, but it is limited by operator dependence, motion, large body habitus, obscuration by gas,^6^,^7^ and oversampling of the posterior gland with undersampling of the apex and anterior gland.^6^–^9^ The more than 50% false positive biopsy results observed are usually from prostatitis and benign prostatic hypertrophy.^10^ In addition, there is a significant false negative rate of at least 30% for clinically significant cancers (defined as Gleason score >7 and volume >0.5 cm^3^).^11^–^13^ Overdiagnosis of clinically insignificant lesions (defined as small cancers with Gleason scores <7) is also problematic.^4^,^14^

### Multiparametric MRI

By virtue of its anatomic and functional information, multiparametric MRI (mpMRI) (i.e. multiple pulse sequence MRI) offers significant advantages over TRUS for lesion detection, characterization, and local staging of prostate cancer. Use of endorectal coils and high-field (i.e. ≥3.0 Tesla) MR equipment is usually preferred due to increased signal-to-noise ratio and image quality.

Anatomic MRI pulse sequences are typically T1- and T2-weighted. The T2-weighted images are relatively high-resolution, showing zonal anatomy of the prostate to advantage. Functional sequences are diffusion-weighted (DWI) and dynamic contrast-enhanced (DCE) imaging. Apparent-diffusion coefficient (ADC) maps are derivatives of DWI measurements. Dynamic contrast-enhanced imaging is dynamic pre- and post-contrast-enhanced gradient echo imaging of the prostate. Most malignant lesions show focal early enhancement due to neovascularity, but this is not specific. Dynamic contrast-enhanced imaging is most useful when T2 and DWI images are indeterminate.

The multiple variables and pulse sequences in MRI are challenging to analyze and synthesize, resulting in a high interobserver variability.^15^,^16^ Fortunately the recently updated Prostate Imaging Reporting and Data System Version 2 (PI-RADS 2.0) is a guide to the performance, interpretation, and reporting of prostate MRI with risk stratification.^17^ This document will soon be accompanied by an atlas (PI-RADS v2 Atlas) that will likely be very useful to assist radiologists in pattern recognition.

A recently reported reader study documents that PI-RADS 2.0 enables prostate cancer detection with average sensitivity of 63%, across all lesions and all readers (including both general radiologists and subspecialists in prostate MRI), with a high index of 74% of specific agreement among readers.^16^ Another recent study from a data warehouse including four million patients in the Chicago area suggested a local increase of 486% in the use of mpMRI for detection and management of prostate cancer from 2013 to 2015.^18^

**Figure 3:**
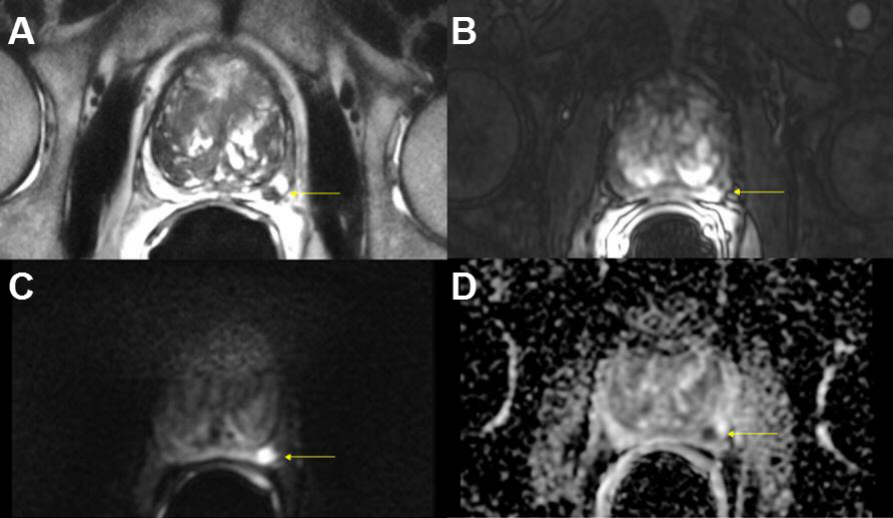
Multiparametric MRI of a Typical Malignant Prostate Nodule in a 61-Year-Old Male Left peripheral zone lesion showing low signal on T2-weighted images (A), early avid enhancement on T1-weighted images after gadolinium injection (B), high signal on b=1400 ms diffusion-weighted images (C), and low signal on ADC map (D).

**Figure 4:**
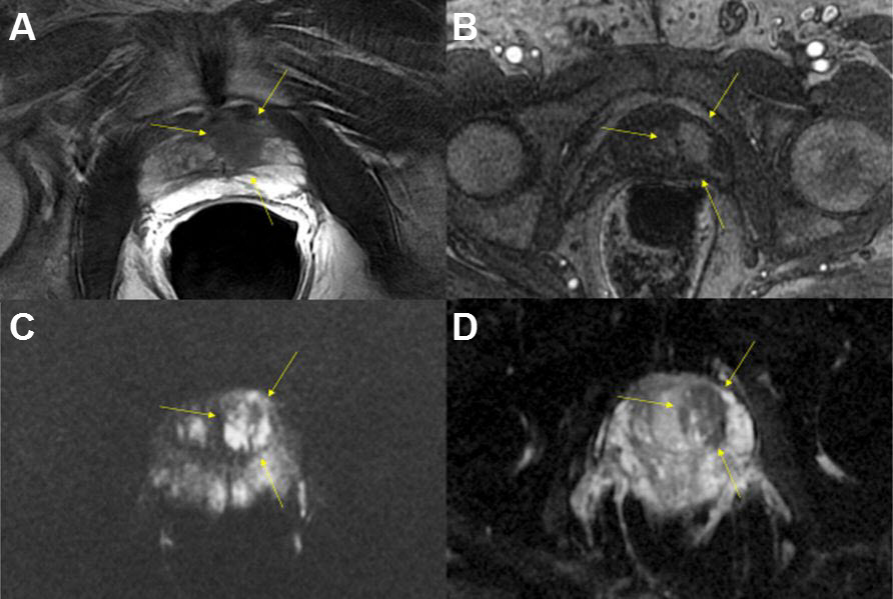
Multiparametric MRI of the Prostate of a 65-Year-Old Man Shown are a transitional zone lesion with low signal on T2-weighted images (A), early avid enhancement on T1-weighted images after gadolinium injection (B), high signal on b=1400 ms diffusion-weighted images (C), and low signal on ADC map (D). Detection of lesions in this location is challenging with transrectal ultrasound, but feasible with mpMR.

Diffusion-weighted imaging sequences are sensitive to random water motion in tissues. Diffusion is typically restricted in tumors compared to normal tissue. High b-value imaging used to make DWI sequences of prostate cancer show high signal in tumor tissue. Apparent-diffusion coefficient maps are used to quantify the restriction of diffusion (dark signal) by measuring at multiple b-values (i.e. gradient amplitudes). Values of ADC are decreased with restricted diffusion, but this is non-specific. (Figures 3 and 4).^19^

### Multiparametric MRI-US Fusion

Multiparametric MRI is more sensitive and specific than TRUS, but it is more expensive, more time-consuming, and more uncomfortable than TRUS, requires non-ferromagnetic equipment, and lacks the real-time feedback of TRUS. In order to optimize the advantages of both modalities, mpMRI-US fusion guided biopsies were developed in which previously acquired MRI scans are mapped onto TRUS using co-registration or fusion techniques that employ electromagnetic sensors.

**Figure 5:**
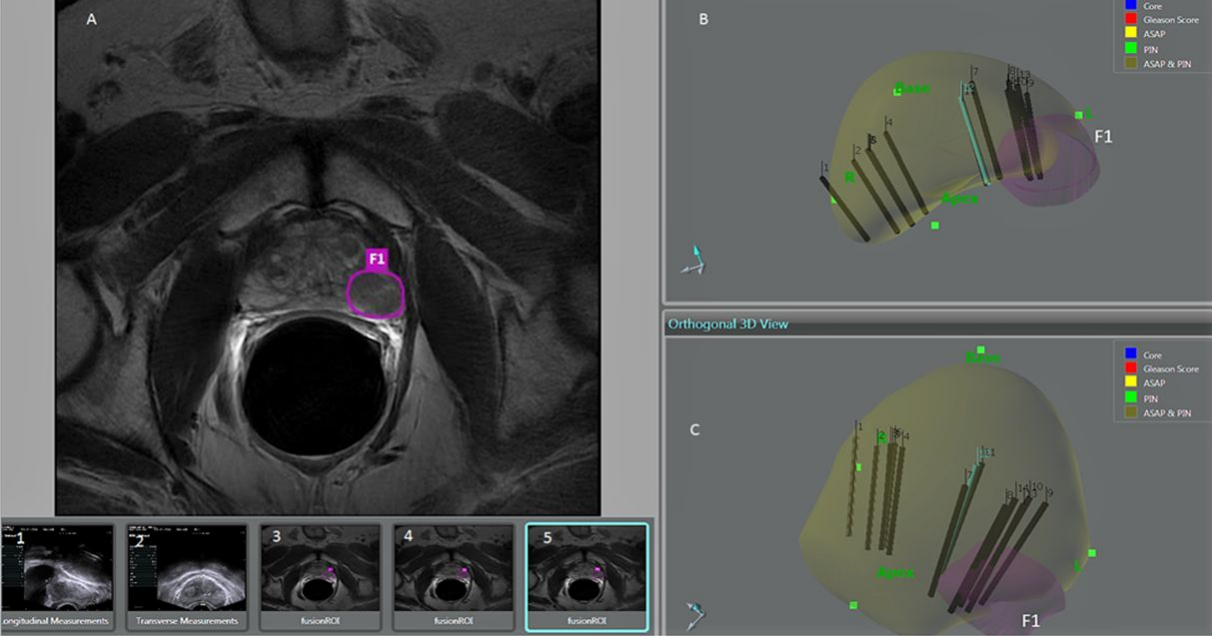
Multiparametric MRI-TRUS Fusion Guided Biopsy in a 62-Year-Old Male The mpMRI-TRUS fusion guided biopsy in a 62-old year male, who had previous negative biopsy one year prior, shows a focal nodule in the left peripheral zone which underwent four core samples revealing Gleason (4+3) 7 in 80% of the specimens. The mpMRI circled region shows a left peripheral zone hypointense lesion (A). Sagittal (B) and coronal (C) depiction from 3D model of mpMRI-TRUS fusion; the left target lesion (F1) is depicted in pink. Sagittal (1) and transverse (2) TRUS images for measurement of the size of the prostate. The mpMRI images show the left peripheral zone lesion circled (3, 4, 5); these images are used to localize the F1 lesion for fusion guided biopsy.

Using technology similar to GPS positioning systems, the latest commercially available devices use three-dimensional (3D) volumetric US acquisitions with either rigid or elastic fusion of the superimposed 3D volumetric MRI. Rigid fusion permits better co-registration between the two modalities, while elastic fusion enables localized corrections for deformations in shape due to positioning, bladder and rectal filling, and different degrees of compression from the endorectal balloon compared with the TRUS probe. Thereafter, significant lesions are mapped and targeted for mpMRI-TRUS fusion guided biopsies (Figure 5).^19^–^26^

Limitations of this technique include imprecise co-registration, inability to compensate for motion or prostate compression causing registration error during biopsy, and requirements for expertise during needle positioning and deployment. The cost of mpMRI-TRUS fusion and its reimbursement, limited availability, variety of fusion equipment and techniques, and lack of uniform guidelines are problematic at present. Harmonizing these two last-mentioned factors will empower a clear assessment of the clinical utility of the technique.^27^,^28^

Multiparametric MRI-TRUS fusion directed biopsy increases the detection rate of clinically significant prostate cancer and decreases that of clinically insignificant cancers. The grading and staging are more accurate than using TRUS guidance alone.^14^,^24^ A meta-analysis of 15 studies with 2,293 patients showed that the median detection rate for clinically significant disease was about 33%, while that of standard TRUS was 24%.^29^ Another meta-analysis of 11 studies and 2,626 patients showed a similar higher cancer detection rate with fusion.^30^ There is still a significant false negative rate with this fusion technique, i.e. as high as 20% in patients when no significant lesions are found on fusion imaging.^31^–^33^ However, a recent report found that only 62 of 1,003 cases (6.2%) were upgraded by systematic TRUS biopsy after fusion biopsy, indicating that fusion biopsy infrequently misses important cases.^27^

## PSMA PET/CT

The PET/CT imaging modality is a well-known hybrid fusion imaging technique that marries together the excellent spatial resolution of computed tomography with functional imaging that highlights metabolic activity in abnormal cells. Whole-body PET/CT has become a mainstay of oncologic imaging because it can localize and quantitate active tumor metabolism at the primary site, in metastases, and in local and distant recurrence, as well as guide tissue sampling and medical decision-making about therapy.

Recently PET/MR hybrid imaging devices have become commercially available, offering the advantages of high specificity of PET with better soft tissue contrast and intrinsic functional MR information compared to PET/CT. However, PET/MR equipment is very expensive, with relatively slow operating speed, and remains to be validated with clinical research.^34^

There are a number of PET probes that are under investigation for prostate cancer imaging, including ^11^C-choline, ^18^F-fluoroethylcholine, ^11^C-methionine, and ^18^F^-^dihydrotestosterone. The specificity needed to differentiate prostate cancer from prostatitis or benign prostatic hypertrophy and the desire for improved sensitivity of PET with its limited resolution are possible barriers to success of these agents.

A very promising imaging biomarker for prostate cancer is PSMA. After ^68^Ga PSMA is injected into the patient, it binds to cells that express PSMA. The antigen is a transmembrane protein that is overexpressed by prostate cancer cells up to 1000-fold compared to normal prostatic tissue.^35^ It is noteworthy that increasingly aggressive prostate cancers have increased expression of PSMA.^36^

However, PSMA is also expressed in other normal tissues such as salivary glands, bladder, pancreas, lung, kidneys, lacrimal glands, liver, spleen, intestines, celiac ganglion, and astrocytes.^37^–^39^ It is also seen in duodenal mucosa, some proximal renal tubule cells, some neuroendocrine cells in colon crypts, and some transitional cell, renal cell, colon, and other carcinomas.^37^ False negative scans have been seen in prostate cancer that has neuroendocrine differentiation, small size lesions, and lesions in close proximity to high physiologic uptake such as the celiac ganglia.^40^

The ^68^Ga PSMA PET/CT (or PET/MR) hybrid modality may provide localization staging of primary prostate cancer, guide biopsy, and even offer future opportunities for focal therapy through theranostics. This agent outperforms ^18^F-choline and ^11^C-choline in primary staging and restaging of prostate cancer.^41^,^42^ Gallium-68 is available using gallium generators which may be located in the imaging department without the need for access to a cyclotron,^43^ and has a relatively short half-life of 68 minutes. It is quickly cleared and has relatively low background activity.^44^

**Figure 6:**
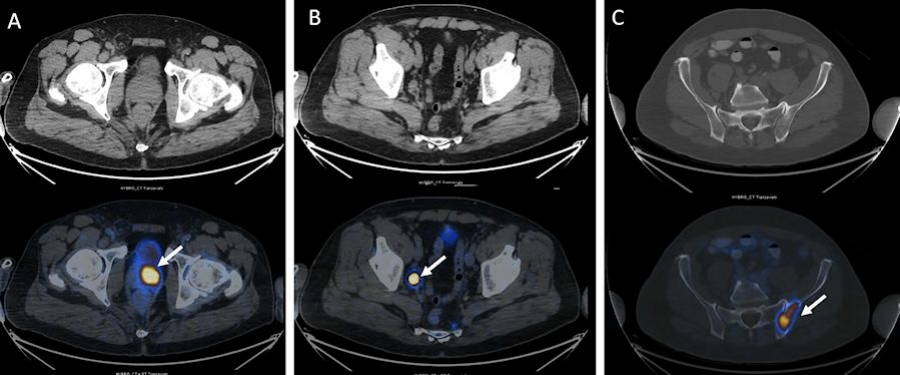
A 59-Year-Old Male with Newly Diagnosed Prostate Cancer (Gleason Score, 7) Staging ^68^Ga PSMA PET/CT scan in a 59-year old male with newly diagnosed prostate cancer (Gleason score, 7) demonstrates pathological PSMA uptake involving most of the left prostate lobe and extending across the midline **(A)**, a right pelvic lymph node metastasis **(B)**, and a left iliac bone metastasis (arrows) **(C)**. PSMA PET is sensitive for detection of micrometastases in nodes less than 1 cm in short axis measurement, as in **(C)**. Additional sites of lymphadenopathy and skeletal metastases were demonstrated on this PSMA PET/CT study (not shown).

There are early reports of accurate primary local staging and improved detection of metastatic lymphadenopathy with high sensitivity and specificity using PSMA PET/CT.^41^,^45^–^50^ [^68^Ga]Gallium PSMA used with PET/CT outperformed standard CT and MRI in one study of 130 patients with a sensitivity of 75%, specificity of 98.9%, and accuracy of 88.5% compared to 43.9%, 85.4%, and 72.3%, respectively, for CT and MRI.^41^ Similar findings were noted in a study of 37 patients with intermediate- or high-risk prostate cancer, with sensitivity of 75% and specificity 96% with the same agent.^51^ Another study of 21 patients showed moderate sensitivity of 67%, specificity of 92%, positive predictive value of 97%, negative predictive value of 42%, and 72% accuracy using a six segment model (Figure 6).^52^

However, there is a false negative rate of PSMA imaging, reportedly 8% of patients in one study.^41^ In another study, two of four negative scans were false negatives.^51^

There is speculation that the negative studies may be due to a saturation effect of uptake from the primary cancer in the prostate gland, or micrometastases below a threshold size for detection, or due to inadequate sample size in one cohort of 30 patients. That same study reported PSMA PET/CT results for detection of nodal metastases with a sensitivity of 33.3%, specificity of 100%, positive predictive value 100%, and negative predictive value 100%.^49^ The importance of detecting micrometastases cannot be overemphasized because nodal involvement affects staging and therefore treatment selection. Moreover, the majority of metastatic prostate cancer nodes measure less than 8 mm, such that CT and MRI based on size criteria are known to be inaccurate.^53^

Now in its infancy, PSMA PET/CT fused imaging will require standards for imaging protocols, reviewing, and reporting that are under development.^54^

### Multimodality Fusion

Multimodality fusion of TRUS, mpMRI, and PSMA PET/CT can be accomplished with co-registration of these multiple data sets using dedicated DICOM (Digital Imaging and Communications in Medicine) software platforms. The potential value of such combined imaging is much greater than the sum of its parts. Initially there will be much attention paid to discriminatory information such as improved detection of clinically significant cancers without overdiagnosis, and detection of extracapsular extension, because these findings change management and therapy.

**Figure 7:**
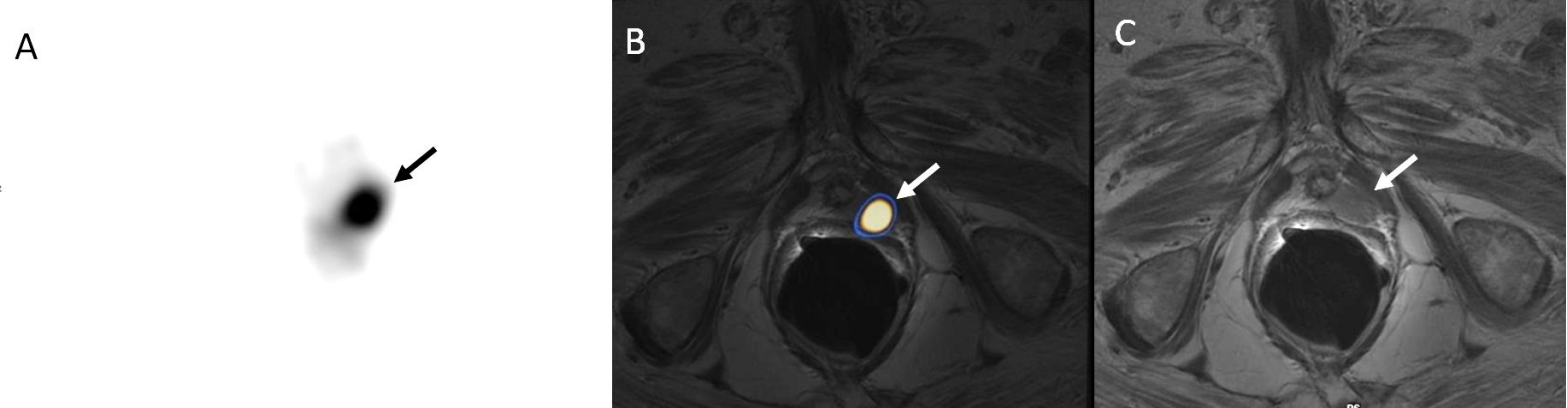
A 66-Year-Old Male with Newly Diagnosed Prostate Cancer (Gleason Score, 7) Transaxial PET slice **(A)** shows focal PSMA activity. Fused images **(B)** of ^68^Ga PSMA PET and (separately performed) MRI **(C)** show the PSMA activity localized to an apical solitary lesion at the left lobe of the prostate gland (arrows).

Imaging with ^68^Ga PSMA PET/CT can augment mpMRI by showing increased activity that is very specific and sensitive for prostate cancer, especially because MRI alone suffers from false positive results and may not detect central gland lesions with the same sensitivity as peripheral ones, as described above (Figure 7).

Multimodality fusion of ^68^Ga PSMA PET/CT with mpMRI could provide complete local staging of prostate cancer, identification of lymphadenopathy, and evaluation of distant metastases unified into a single imaging display. Add to this the easy access and flexibility of TRUS, also fused with the above, and the advantage of optimized targeted biopsies for primary cancer staging is clear. The opportunity immediately to locate, stage, and treat the tumor is attractive, especially because medical decision-making is streamlined when there is a “one-stop-shop.”

Multiple registration tasks are required for our research: mpMRI with ^68^Ga PSMA PET/CT, followed by co-registration with real-time TRUS for targeted biopsies. Thereafter, focal therapy guided by the multimodality fusion imaging with HiFUS for focal therapy in organ-confined disease is planned. The ideal reference standard is whole mount prostate pathology sections with histologic mapping onto fusion imaging in patients undergoing radical prostatectomy. We will also track localized needle biopsy results in patients excluded from radical surgery.

### Focal Therapy with HiFUS

**Figure 8:**
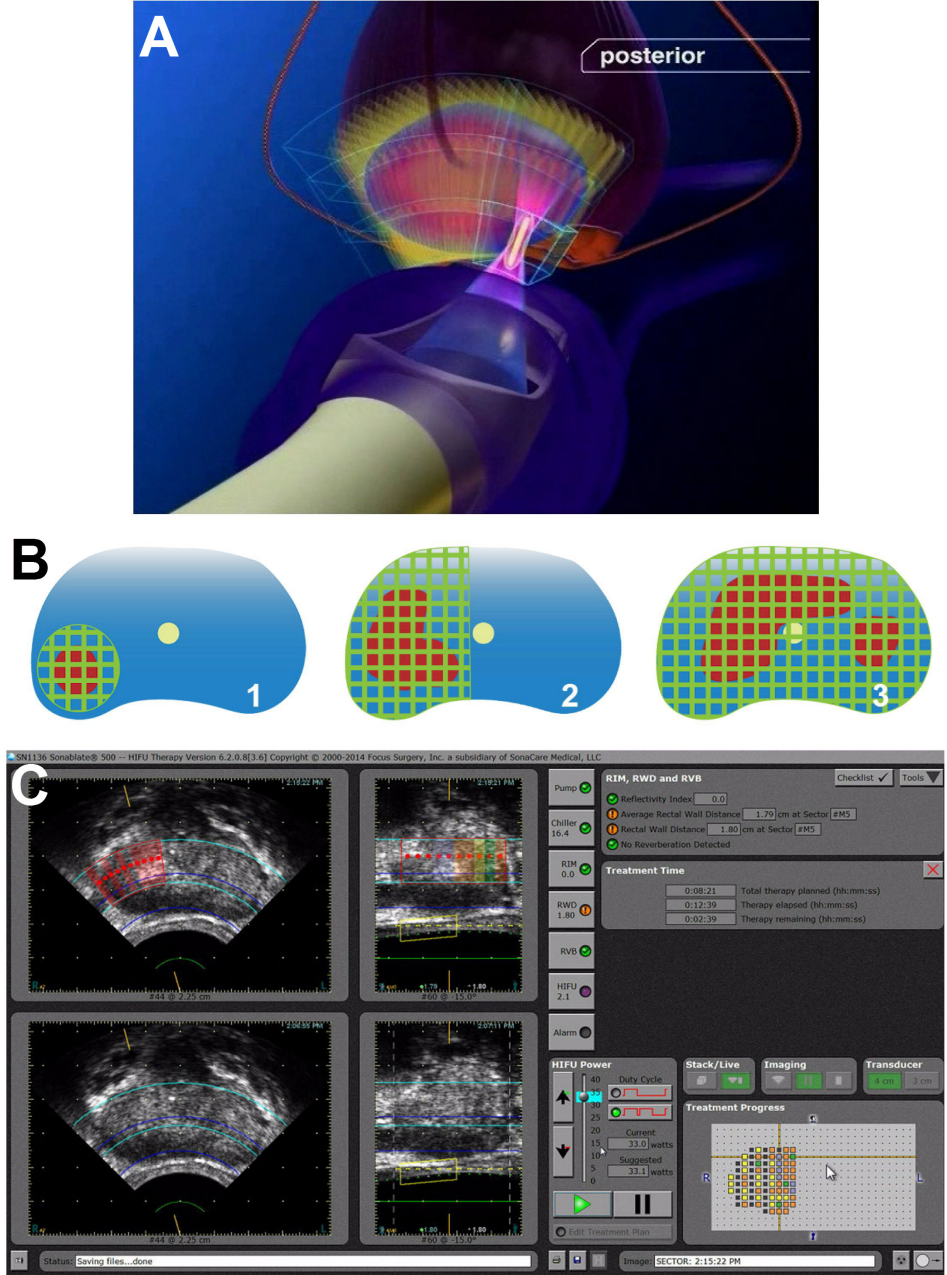
Focal Therapy with HiFUS **(A)** Rendering of focal therapy using HiFUS for a left posterior dominant nodule. (**B)** Depiction of HiFUS prostate ablations. Lesions are depicted in red, and cross-hatched green lines represent the treated volume: (1) focal treatment of prostate; (2) hemi-ablation of prostate; (3) whole gland treatment of prostate. **(C)** Display console during planning and execution of right focal ablation of prostate cancer. Red-shaded area represents the targeted treatment volume. All panels reproduced with permission from SONACARE Inc.

Multimodality imaging has strategic value in planning and guiding focal prostate cancer therapy. Successful focal therapy necessarily involves accurate definition of organ-confined malignancies followed by their complete eradication while sparing surrounding normal tissue. Various methods for focal therapy include high-intensity focused ultrasound (HiFUS), radiofrequency ablation, cryoablation, and electroporation (Figure 8).

High-intensity focused US uses a sharply focused delivery of high thermal energy to heat and destroy the intended target without ionizing radiation.^55^,^56^ Complications have been reported from HiFUS such as bladder outlet obstruction, impotence, urethral stricture, and urinary incontinence.^57^ However, the potential advantages include reduced morbidity, lower complication rate (impotence and incontinence), and rapid recovery compared to radical surgery. A recent meta-analysis of 13 studies in 346 patients undergoing HiFUS for focal therapy with median follow-up of 12 months showed probability of transition to secondary local treatment was 7.8%, disease-specific survival was 100%, pad-free continence was achieved in 100%, and impotence occurred in 11.4%.^58^

Several countries have approval for the use of HiFUS devices for human use including the United States, Canada, Australia, and others, with various clinical trials underway. Because there is a need for long-term follow-up and standardization of treatment protocols in using HiFUS for focal therapy,^59^ this procedure is experimental. It has mostly been used in clinical trials, or in men with comorbidities who have low- to intermediate-risk disease and decline active surveillance or radiation treatment.^57^,^60^

## FUTURE DIRECTIONS

We hypothesize that multimodality fusion will accurately target clinically significant lesions, improve performance in lesions with Gleason heterogeneity, allow documentation of biopsy locations, and facilitate focal therapy planning and performance. Precision in treating targeted tissues with minimally invasive techniques is the goal. There is a need for speedy fusion with stable registration and accuracy in lesion ablation for successful diagnosis and treatment. Single-center pioneering studies for multimodality prostate cancer imaging and focal therapy, such as the one our institution embarks upon now, may be followed by well-designed prospective multi-center trials.

How can we process the enormous data sets from multimodality imaging? As technical advances continue, we will be pushed to our human limits to process these giant data sets. The answer lies in machine learning using texture analysis with computer-aided detection and diagnosis. Mathematical models and algorithms are a necessity to manage the imaging and clinical data for personalized care. Validation of these models is essential if we are to achieve accurate lesion characterization, risk stratification, staging, treatment selection, surveillance, and evaluation of response to therapy.

This unified approach will grow as we move closer and closer to the holy grail: the ultimate “find it and fix it” using a single theranostic agent that will simultaneously show and kill cancer. Early research with new agents under development shows promise.^61^,^62^

## PREOPERATIVE STAGING

Magnetic resonance imaging provides the most useful information of the local extent of tumor in prostate cancer, but MRI underestimates lymph node involvement due to the use of short-axis node measurements of 1.0 cm as the criterion for positive nodes. Micrometastases (nodes smaller than 1.0 cm) can be missed.^53^ A more accurate method of detecting nodal metastases is desirable to achieve accurate preoperative staging.

In patients who do not undergo radical prostatectomy, additional biopsy procedures are essential when extraprostatic tumor may be present to document pathologic stage and triage patients to appropriate therapy. For evaluation of metastases, traditionally bone scan with technetium-99m has been sensitive for detection of bone lesions, while CT or MRI can be accurate for identification of visceral metastases.^63^

It is noteworthy that PSMA PET/CT also holds great promise as an agent for identification of nodal and bony metastases from prostate cancer. Thus PSMA PET/CT with multimodality fusion imaging may provide not only local staging and targeted prostate biopsies but also may identify extraprostatic sites for selected biopsies to confirm metastases.^41^ In cases with documented metastases, inappropriate focal therapy can be avoided.

## SUMMARY

There are many challenges to overcome. Regulatory requirements, funding for expensive research and development of experimental agents, and undefined reimbursement are but a few. Nevertheless, multimodality imaging and focal therapy for prostate cancer based on future well designed multi-center prospective clinical trials will probably inform clinical decision-making for decades to come.

## Abbreviations

3D: three-dimensional
ADC: apparent-diffusion coefficient
CT: computed tomography
DCE: dynamic contrast-enhanced
DICOM: Digital Imaging and Communications in Medicine
DWI: diffusion-weighted imaging
HiFUS: high-intensity focused ultrasound
mpMRI: multiparametric MRI
MRI: magnetic resonance imaging
PET: positron emission tomography
PI-RADS 2.0: Prostate Imaging Reporting and Data System Version 2
PSMA: prostate-specific membrane antigen
TRUS: transrectal ultrasound
US: ultrasound.
